# Managing the green proteomes for the next decade of plant research

**DOI:** 10.3389/fpls.2013.00501

**Published:** 2013-12-16

**Authors:** Andrew W. Carroll, Hiren J. Joshi, Joshua L. Heazlewood

**Affiliations:** ^1^Department of Cellular and Molecular Medicine, Copenhagen Center for Glycomics, University of CopenhagenCopenhagen, Denmark; ^2^Physical Biosciences Division and Joint BioEnergy Institute, Lawrence Berkeley National LaboratoryBerkeley, CA, USA

**Keywords:** proteomics, informatics, database, phosphorylation, proteogenomic, subcellular

For the past decade the field of proteomics has transitioned from a highly specialized research area into a conventional technique widely employed by plant biologists. This approach now encompasses basic protein identification to advanced comparative studies. The result has been an abundance of proteomics data, often not readily available to the research community (Heazlewood, 2011). This has resulted in the creation of numerous proteomic resources which are often referred to as boutique databases. Generally, these sites exist outside the traditional community driven centralized repositories. While the geographic location of web-based resources is somewhat inconsequential, it can highlight active regions of plant proteomic-based research. The intention of this Research Topic on Plant Proteomic Resources is to collect articles focusing on these resources and provide an overview of current online plant proteomic portals.

Plant proteomic resources are often integrative and comprise collections of diverse ‘omics information to support the proteomic data. A good example of this is the GabiPD portal (Usadel et al., 2012), the website is a gateway for the German plant community to codify and unite various research programs at one site. More focused resources such as pep2pro was constructed to support large-scale proteomic surveys in the model plant Arabidopsis (Hirsch-Hoffmann et al., 2012). The pep2pro repository employs a unique workflow to match spectral data directly against the Arabidopsis genome. Although techniques for arraying proteins by 2-DE have been employed for decades, the GelMap portal links proteomic-based identifications with gel electrophoresis maps (Senkler and Braun, 2012). The GelMap resource provides annotated two-dimensional arrays of proteins from a range of sample types.

The application of proteomics to characterize organelles were some of the first large-scale surveys in plants. The AT_CHLORO database represents the most extensive analysis of the chloroplast from the model plant Arabidopsis (Bruley et al., 2012). This resource provides a compendium of proteins identified in the chloroplast and contains information on its sub-compartments e.g., thylakoid. Organelle proteome databases such as AT_CHLORO comprised many of the early online plant proteomic databases including the mitochondrion (Heazlewood and Millar, 2005) and the peroxisome (Reumann et al., 2004). The latter was recently used to develop a new resource, PredPlantPTS1, which predicts whether a protein will localize to the peroxisome (Reumann et al., 2012). The SUBcellular Arabidopsis database (SUBA) contains data from most subcellular proteomic surveys in Arabidopsis (Tanz et al., 2013). A similarly focused resource, the Plant Protein DataBase (PPDB) also deals with subcellular proteomics but also encompasses other plant species (Sun et al., 2009). Although the latter two resources are not part of this collection, data housed by these repositories are available through the MASCP Gator, a portal designed to aggregate Arabidopsis proteomic data for the community. The MASCP Gator interface was developed to provide a mechanism for proteomic data visualization from multiple data sources (Mann et al., 2013).

The model plant Arabidopsis dominates the plant proteomic resource landscape, but as genomic information in other plant species becomes available, databases for other species have been established. The rice RNA-binding protein resource provides a curated collection of over 250 experimentally identified RNA interacting proteins from rice (Doroshenk et al., 2012), providing functional annotation, expression, and phylogenetic relationships. Large-scale developmental and organ specific analyses of the rice proteome has now, also been conducted. These data are available through the rice proteogenomics database (OryzaPG-DB) which provides a visual relationship between the genome and the identified proteome (Helmy et al., 2012). The Soybean Proteome Database (SPD) initially focused on curating proteins that were responsive to flooding (Ohyanagi et al., 2012), but it now includes a host of 2-DE arrayed organelle proteomes, expression information and information on other stress induced proteins from this important leguminous crop.

Seed development represents a major agricultural focus for plant researchers and as such, this developmental process has been extensively targeted by proteomic surveys. The seed proteome web portal provides an extensive collection of data, including quantitative information on proteins involved in seed development (Galland et al., 2012). As is the case with the Seeds of Chernobyl resource, which highlights a different aspect of seed development in plants, namely cataloging the effects of ionizing radiation on seed maturation and development (Klubicova et al., 2012).

Post-translational modifications (PTMs) often represent the functional state of a protein and are a significant objective for many proteomic studies. A number of resources have been developed to interact with these phosphoproteomic datasets. Initial phosphoproteomic surveys involved Arabidopsis and one of the first phosphorylation-based databases created in any species was PhosPhAt (Arsova and Schulze, 2012). The resource contains many thousands of experimentally identified sites available in the literature. The expansion of phosphoproteomic surveys outside Arabidopsis has resulted in the creation of two further resources, the P^3^DB database houses tens of thousands of phosphopeptides from six plant species. The collection of such an array of data by P^3^DB led to the development of Musite, a utility that predicts phosphorylation sites in plant proteins (Yao et al., 2012). Lastly, the Medicago PhosphoProtein Database houses data from a recent large-scale phosphoproteomic analysis of this model legume plant (Rose et al., 2012).

The proteomics community has created an array of online tools that can be used to support various technical approaches in mass spectrometry. The MRMaid utility was designed to facilitate the selection of peptides for targeted proteomic analyses (Fan et al., 2012). The tool leverages plant spectral information housed in the PRIDE repository (Vizcaino et al., 2013) to assist in the selection of protein specific peptides for multiple reaction monitoring (MRM) of plant samples. In a similar vein, the ProMEX resource enables newly collected tandem mass spectrometry data to be queried against previously matched experimental spectra (Wienkoop et al., 2012). Spectral matching process provides real world context as tandem mass spectra generally do not produce evenly distributed fragment ions.

The range of proteome resources highlighted in this Research Topic reflect the diversity of proteomic-based applications in plant sciences. The principle objective for many these research groups has been focused on cataloguing or collecting data in an effort to capture information. Indeed, the creation of many data portals likely reflects an attempt to make sense of one's own data. This collection highlights the diversity and range of plant proteomic resources and utilities available to the plant research community.
